# SALMON: Survival Analysis Learning With Multi-Omics Neural Networks on Breast Cancer

**DOI:** 10.3389/fgene.2019.00166

**Published:** 2019-03-08

**Authors:** Zhi Huang, Xiaohui Zhan, Shunian Xiang, Travis S. Johnson, Bryan Helm, Christina Y. Yu, Jie Zhang, Paul Salama, Maher Rizkalla, Zhi Han, Kun Huang

**Affiliations:** ^1^School of Electrical and Computer Engineering, Purdue University, West Lafayette, IN, United States; ^2^Department of Medicine, Indiana University School of Medicine, Indianapolis, IN, United States; ^3^Department of Electrical and Computer Engineering, Indiana University-Purdue University Indianapolis, Indianapolis, IN, United States; ^4^National-Regional Key Technology Engineering Laboratory for Medical Ultrasound, Guangdong Key Laboratory for Biomedical Measurements and Ultrasound Imaging, School of Biomedical Engineering, Health Science Center, Shenzhen University, Shenzhen, China; ^5^Department of Medical and Molecular Genetics, Indiana University School of Medicine, Indianapolis, IN, United States; ^6^Department of Biomedical Informatics, The Ohio State University, Columbus, OH, United States; ^7^Regenstrief Institute, Indianapolis, IN, United States

**Keywords:** deep Learning, co-expression analysis, survival prognosis, breast cancer, multi-omics, neural networks, cox regression

## Abstract

Improved cancer prognosis is a central goal for precision health medicine. Though many models can predict differential survival from data, there is a strong need for sophisticated algorithms that can aggregate and filter relevant predictors from increasingly complex data inputs. In turn, these models should provide deeper insight into which types of data are most relevant to improve prognosis. Deep Learning-based neural networks offer a potential solution for both problems because they are highly flexible and account for data complexity in a non-linear fashion. In this study, we implement Deep Learning-based networks to determine how gene expression data predicts Cox regression survival in breast cancer. We accomplish this through an algorithm called SALMON (Survival Analysis Learning with Multi-Omics Neural Networks), which aggregates and simplifies gene expression data and cancer biomarkers to enable prognosis prediction. The results revealed improved performance when more omics data were used in model construction. Rather than use raw gene expression values as model inputs, we innovatively use eigengene modules from the result of gene co-expression network analysis. The corresponding high impact co-expression modules and other omics data are identified by feature selection technique, then examined by conducting enrichment analysis and exploiting biological functions, escalated the interpretation of input feature from gene level to co-expression modules level. Our study shows the feasibility of discovering breast cancer related co-expression modules, sketch a blueprint of future endeavors on Deep Learning-based survival analysis. SALMON source code is available at https://github.com/huangzhii/SALMON/.

## Background and Introduction

There is a strong need to identify effective prognostic biomarkers to help optimize and personalize treatment (Liu et al., 2016). Among cancers, breast invasive carcinoma is one of the most heterogeneous cancers with distinct prognoses based on morphological, phenological, and molecular stratifications (Nagini, 2017; Wu et al., 2017). Breast invasive carcinoma patients have a 77% survival rate after 5 years and 44% survival rate after 15 years (Pereira et al., 2016), so developing accurate prognostic models could significantly improve risk stratification after diagnosis.

Recent Deep Learning-based approaches have been widely applied to Computational Biology and Bioinformatics (Huang et al., 2017; Zhang et al., 2018b). The advantages of learning non-linear functions and retrieving lower dimensional representation (Ching et al., 2018) reveal advances of Deep Learning models. The application of survival prognosis that incorporates Cox proportional hazards regression with a single transcriptomic dataset (Ching et al., 2018; Katzman et al., 2018; Shao et al., 2018) and with multi-omics data (Chaudhary et al., 2018; Poirion et al., 2018; Ramazzotti et al., 2018; Sun et al., 2018; Zhang et al., 2018a) is of major interest in precision health.

For these reasons, we integrate multi-omics data with Deep Learning-based survival prognosis models. While most contemporary approaches incorporate one or few types of omics data, such as mRNA-seq data and miRNA-seq data (Gupta et al., 2015; Nassar et al., 2017), we propose that integrating more diverse data may lead to improved modeling—especially when driven by machine learning. Moreover, classic cancer biomarkers can often stratify patients into risk groups, and these too should be integrated when available. Specifically, copy number burden (CNB) and tumor mutation burden (TMB) are important for predicting tumor progression (Marshall et al., 2017; Thomas et al., 2018) and immunotherapy (Birkbak et al., 2013; Chalmers et al., 2017; Goodman et al., 2017). Other demographical and clinical information such as diagnosis age, estrogen receptors (ER) status, progesterone receptors (PR) status should also be considered during model construction. One of the challenges for such diverse data is high-dimensionality.

Most Deep Learning approaches employ neural networks (multilayer perceptron) with huge numbers of parameters to be optimized. Optimizing such large sets of parameters with limited patient samples tends to introduce overfitting that renders the models ineffective. In this paper, we advocate the use of eigengene matrices instead of original mRNA-seq and miRNA-seq data derived from co-expression analysis with R package “lmQCM.” Using neural network architecture, multi-omics data, and the Cox proportional hazards model, we develop our model called SALMON (Survival Analysis Learning with Multi-Omics Neural Networks). SALMON adopts co-expression modules as input, namely, the eigengene matrix derived from co-expression network analysis. It greatly reduces the dimension of the original feature space addressing the “curse of dimensionality” and increases the robustness and learnability of the model. This novel technique was not adopted by any other Deep Learning-based survival prognosis model such as Cox-nnet (Ching et al., 2018).

**Figure 1:**
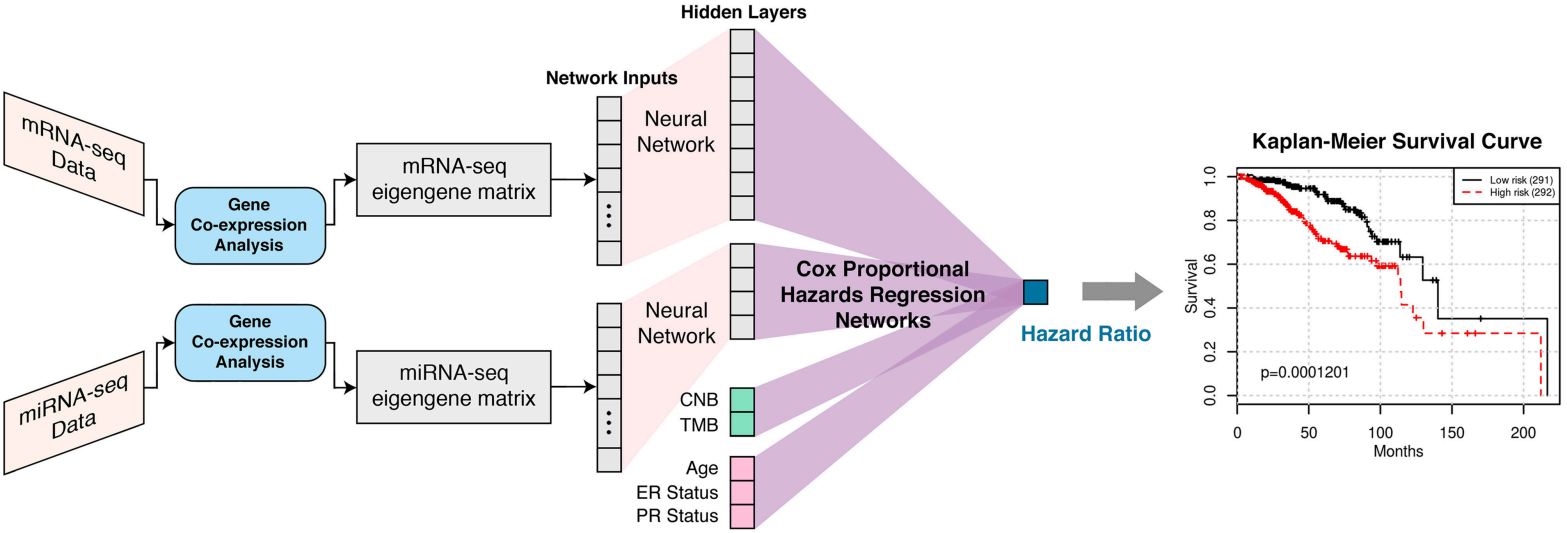
SALMON (Survival Analysis Learning with Multi-Omics Neural Networks) architecture with the implementation of Cox proportional hazards regression networks. Co-expression modules (eigengene matrices) are the inputs to the SALMON. Number of the hidden layers and dimensions of hidden layers can also be fine-tuned (not included in this paper). The output is the hazard ratios which can be interpreted as the relative risks of patients.

SALMON is trained on co-expression module eigengenes instead of gene expressions and thus we were able to investigate co-expression modules contribution to the hazard ratio (Figure 1). These gene co-expression modules contained individual genes from the initial lmQCM gene co-expression network analysis. Genes from modules that highly contributed to the hazard ratio were further evaluated with gene enrichment analysis to confirm certain gene regulations and biological processes. These biological findings confirm the validity of our models and provide insight into the complex regulatory relationships at work in breast invasive carcinoma.

## Materials and Methods

### Datasets and Study Design

In this experiment, we analyzed 583 female breast invasive carcinoma (BRCA) patients which had five omics data types including gene expression data (illuminahiseq_rnaseqv2-RSEM_genes_normalized) and miRNA data (illuminahiseq_mirnaseq-miR_gene_expression) from Broad GDAC Firehose (https://gdac.broadinstitute.org/), copy number burden (CNB) was measured by total Kb length and the data (broad.mit.edu_PANCAN_Genome_Wide_SNP_6_whitelisted.seg) was provided from Pan-Cancer Atlas (PanCanAtlas) Initiative (https://gdc.cancer.gov/about-data/publications/pancanatlas). Tumor mutation burden (TMB) was calculated by the total number of mutated genes based on MAF files (Mutation_Packager_Oncotated_Calls) from Broad GDAC Firehose. Demographical and clinical information (diagnosis age, Estrogen Receptor (ER) status, Progesterone Receptor (PR) status) and overall survival (OS) events and months were collected from cBioPortal (http://www.cbioportal.org/). HER2 status was not considered in this article because of insufficient data. Table 1 shows the statistical information of this patient cohort.

**Table 1:** Demographical and clinical characteristics of 583 female breast invasive carcinoma (BRCA) patients.

**mRNA size**		**miRNA size**		**OS Months**		**Diagnostic age**		**ER positive ratio**	**PR positive ratio**
**Original**	**Co-expression module**	**Original**	**Co-expression module**	**Median**	**Range**	**Median**	**Range**		
13,132	57	530	12	31.70	0.00–216.59	57	26–90	76.16%	67.41%

*mRNA and miRNA stand for mRNA-seq data and miRNA-seq data. OS stands for overall survival. The status of ER and PR were derived from IHC (immunohistochemistry). All clinical information was collected from cBioPortal*.

We performed 5-fold cross-validation on the dataset. In each fold, 80% of the data were used for model training and 20% of the data were used for model testing. mRNA and miRNA data were pre-processed by TSUNAMI online analysis suite (https://apps.medgen.iupui.edu/rsc/tsunami/). The pre-processing steps are 2-fold: It firstly removed genes with lowest 20% of mean expression values shared by all patients. Then it removed genes with lowest 20% of expression values' variance. These pre-processing steps were necessary to ensure the robustness for the downstream correlational computation in gene co-expression module analysis step.

### Gene Co-expression Module Analysis

Instead of feeding mRNA-seq and miRNA-seq data to the neural networks and analyzing results at the gene level, we used eigengene matrices of gene co-expression modules obtained from lmQCM algorithm (Zhang and Huang, 2014) as the input to the SALMON algorithm. This reduced 99.46% of input features and greatly reduced the number of parameters in the neural networks. Using eigengenes as features can be considered as bias/variance (error/complexity) trade-off in machine learning (Weigend et al., 1990; Geman et al., 1992), which simplifies the networks significantly. The total number of neural network weights to be learned was then narrowed down from 107193 to 521, ensuring the robustness of the learning process and alleviate the overfitting issue (Caruana et al., 2001; Schmidhuber, 2015).

There are many gene co-expression network analysis packages, such as the R package for weighted correlation network analysis (WGCNA) (Langfelder and Horvath, 2008) and local maximal Quasi-Clique Merger (lmQCM) (Zhang and Huang, 2014), which can discover densely connected gene modules across samples/patients. Co-expression network analyses are used increasingly to reveal latent gene-gene interactions, biomarkers and novel gene functions (Horvath et al., 2012; Chandran et al., 2016; Han et al., 2016, 2017; Zhang and Huang, 2017; Xiang et al., 2018). Comparing to WGCNA, weight normalization process in lmQCM was inspired by the spectral clustering (Ng et al., 2002) in machine learning. With efficient implementation of the revision from eQCM (edge-covering quasi-clique merger) algorithm (Xiang et al., 2012), lmQCM allowed module overlap, mining smaller densely co-expressed modules, and thus was adopted in this article. The generally smaller size of mined modules can also generate more meaningful gene ontology (GO) enrichment results (Zhang et al., 2012, 2013, 2016; Shroff et al., 2016; Cheng et al., 2017). The implementation was performed on TSUNAMI. For mRNA-seq data, we set lmQCM parameters γ = 0.7, λ = 1, *t* = 1, β = 0.4, minimum size of cluster = 10, and adopted Spearman's rank correlation coefficient (Mukaka, 2012) to calculate gene-wised correlations. The parameters setting of miRNA-seq data were the same except γ = 0.4, β = 0.6, and minimum size of cluster = 4.

After calculating gene co-expression modules with lmQCM, eigengene matrices were then determined. The eigengene matrix is the expression values of each gene co-expression module summarized into the first principal component using singular value decomposition (SVD) (Golub and Reinsch, 1970). With the first right-singular vector of each module as the summarized expression values, it projects co-expressed genes to 1-D space and thus can be treated as the “super gene.” In our experiment with breast invasive carcinoma, an eigengene matrix with 57 dimensions was derived from mRNA-seq data and an eigengene matrix with 12 dimensions was also derived from miRNA-seq data. Details of co-expression modules and eigengene matrices we derived for this paper are available in Supplementary files. These eigengene matrices were treated as the substitution of the original expression inputs.

### Neural Networks Design, Architecture, and Evaluation Metric

SALMON was designed and implemented in PyTorch 1.0. mRNA-seq and miRNA-seq eigengene matrices were firstly connected to hidden layers with dimensions 8 and 4, respectively, then connected to the final output (hazard ratio) with Cox proportional hazards regression networks. Alternatively, CNB, TMB, and demographical and clinical information (diagnosis age, ER status, PR status) had no hidden layer and were connected to final output directly as covariates. This architecture was explained graphically in Figure 1. The rationale behind this network architecture instead of using simple fully connected networks such as Cox-nnet (Ching et al., 2018) was by assuming (1) each omics type affects the hazard ratio independently; (2) downscale eigengene matrices by hidden layers can force multi-omics data contributed to hazard ratios in a relatively equal scale at Cox proportional hazards regression networks part.

SALMON adopts Adaptive moment estimation (Adam) optimizer (Kingma and Ba, 2015). We set the number of epochs = 100 with fine-tuned learning rates for each 5-folds cross-validation experiments. LASSO (least absolute shrinkage and selection operator) regularization (Santosa and Symes, 1986) is applied to the networks. Sigmoid activation function is also applied right after each forward propagation and Cox proportional hazards regression networks. The Sigmoid function

(1) $$\begin{array}{r} sigmoid\left(x\right)=\frac{1}{1+e^{-x}} \end{array}$$

forces the output range be within 0 to 1, introduces non-linearity to the system. In this model, we set the batch size = 64, and the batch normalization was not adopted. The number of the hidden layers and dimensions of hidden layers can be fine-tuned, in this paper, single hidden layers were attached to transcriptomic data with size = 8 for mRNA-seq modules, and size = 4 for miRNA-seq modules.

#### Cox Proportional Hazards Regression Networks

Our algorithm SALMON, integrated Cox proportional hazards model, differs from previous work (Ma and Zhang, 2018; Sun et al., 2018) which use survival status (living or deceased) in a binary classification problem. In contrast, we also took survival times (overall survival months) into account denoted as *Y*_*i*_ and made our neural networks into a Cox regression learning task. Maximum likelihood estimation (MLE) is then applied to the log partial likelihood

(2) $$\begin{array}{r} \ell \left(\beta \right)=\sum_{i:C_{i}=1}\left(\sum_{k=1}^{K}\beta_{k}X_{ik}-\log\left(\sum_{j:Y_{j}\geq Y_{i}}\exp(\sum_{k=1}^{K}\beta_{k}X_{ik})\right)\right) \end{array}$$

where β are the parameters to be estimated. *C*_*i*_ = 1 indicates the occurrence of the death events for patient *i* with *K*-dimensional input vector *X*_*i*_.

#### Objective Function

Based on Cox proportional hazards regression networks we formulized the objective function of neural networks as:

(3) $$\begin{array}{r} \hat{\Theta }=argmin_{\Theta }\{\sum_{i:C_{i}=1}(\sum_{k=1}^{K}\beta_{k}X_{ik}\\ -\log\left(\sum_{j:Y_{j}\geq Y_{i}}\exp(\sum_{k=1}^{K}\beta_{k}X_{ik})\right))+\lambda {\left\|\Theta \right\|}_{1}\} \end{array}$$

where Θ are the entire network weights (including β) to be optimized via back-propagation, λ is the weight multiplier of LASSO regularization. We set λ = 1 × 10^−5^ in the experiments.

#### Evaluation Metric

Concordance index (Steck et al., 2007), valued from 0 to 1, is used in this article as the evaluation metric of survival prognosis. It is widely adopted to evaluate the performances of survival prognosis models (Ching et al., 2018; Katzman et al., 2018) and is equivalent to the area under the ROC curve (AUC) (Bradley, 1997), which measures the model's distinguishability between living and deceased groups. A concordance index = 0.5 indicates the model makes ineffective prediction. A higher concordance index > 0.5 indicates a better survival prognosis model. For breast invasive carcinoma cancer, we consider a concordance index > 0.7 indicates a good model performance.

### Survival Analysis

Survival analysis with log-rank test (Mantel, 1966) is used to inspect the performances of SALMON on 5-folds cross-validation testing sets. The Kaplan-Meier survival curves are generated by dichotomizing all testing patients to low risk and high risk groups via the median hazard ratio. The corresponding log-rank *p*-value implies the ability of the model to differentiate two risk groups. Lower *p*-values convey better model performances.

### Gene Ontology and Functional Enrichment Analysis

Co-expression modules generated by lmQCM are then exported to ToppGene Suite (Chen et al., 2009) (https://toppgene.cchmc.org/) and Enrichr (Kuleshov et al., 2016) (http://amp.pharm.mssm.edu/Enrichr/). Using ToppGene, we performed functional analysis including Gene ontology (GO) and cytoband analysis. The false discovery rate (FDR) < 0.05 and FDR < 1.0 were considered to be significantly enriched for GO analysis and cytoband analysis, respectively. Human Gene Atlas [up regulated genes in human tissues from BioGPS (http://biogps.org)] and ARCHS4 tissues were also investigated for some certain co-expression modules by Enrichr.

## Results

The experiments were performed with six different combinations of multi-omics data as input sources, they are: (i) mRNA-seq data (mRNA) (57 features); (ii) miRNA-seq data (miRNA) (12 features); (iii) integration of mRNA and miRNA (69 features); (iv) integration of mRNA, miRNA, copy number burden (CNB), and tumor mutation burden (TMB) (71 features); (v) integration of mRNA, miRNA, and demographical and clinical (diagnosis age, ER status, PR status) data (72 features); (vi) integration of mRNA, miRNA, CNB, TMB, and demographical and clinical (diagnosis age, ER status, PR status) data (74 features). Where both RNA-seq co-expression modules are required for all integrative combinations. The SALMON model architecture from Figure 1 removed certain network substructures which not been used and performed 5-folds cross-validation with 583 patients. Concordance index was used to evaluate the performances. SALMON was then compared to several other survival prognosis algorithm Cox-nnet (Ching et al., 2018), DeepSurv (Katzman et al., 2018), generalized linear model with Cox regression (GLMNET) (Friedman et al., 2010), and RSF (Ishwaran et al., 2008) with all omics data fed in. Since their Cox regression model didn't take multi-omics data sources into consideration, we modified their original framework to integrate multi-omics data (with co-expression modules) altogether as single input vector. The feature importance of all 74 covariates were also investigated by repeated feature deletion, then ranked by the median of decreased concordance index, proved and revealed certain biological interpretations.

### Integrating Multi-Omics Features Increased the Performances

**Figure 2:**
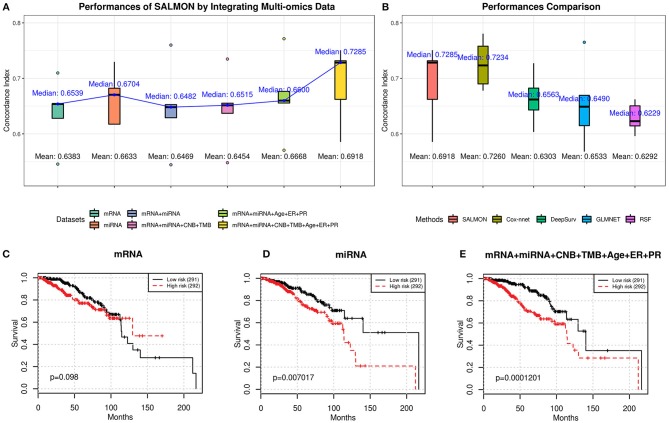
**(A)** Performances of SALMON with multi-omics data integrated in terms of concordance index. **(B)** Performance comparison between SALMON and the modified Cox-nnet, DeepSurv, GLMNET, and RSF in terms of concordance index with all omics data used for learning. **(C–E)** Kaplan-Meier plot of survival prognosis. Hazard ratios were derived from all five testing sets. Log-rank test was used to find the corresponding p-value with low risk and high risk groups dichotomized by the median hazard ratio. Omics data used for training and testing: **(C)** mRNA-seq data (mRNA); **(D)** miRNA-seq data (miRNA); **(E)** integration of mRNA, miRNA, CNB, TMB, and demographical & clinical (diagnosis age, ER status, PR status) data. All other combinations of multi-omics results are in Figure S1.

From Figure 2A, we observed an upward trend on median/mean concordance indices with more omics data are integrated. Integrating all omics data (74 features) gave the optimal performances (concordance index: median = 0.7285; mean = 0.6918). Next, all hazard ratios from 5-folds testing sets were concatenated and performed the log-rank test (Mantel, 1966) as shown in Figures 2C–E and Figure S1. Another feature set without transcriptomics data was also considered as reference (5 features containing CNB, TMB, and demographical and clinical features) with median concordance index = 0.6949 and the Kaplan-Meier plot was shown in Figure S1F (log-rank test *p*-value = 3.67E-03). We found that integrating all omics data (Figure 2E) gave the most significant *p*-value (1.201E-04) with respect to the log-rank test, proving that integrating more multi-omics data to SALMON can enhance the prediction.

We further performed pairwise paired *t*-test to the resulting concordance indices. As shown in Table 2, a negative t-statistic implied that the set 1 is lower than set 2. This concludes that integrating more omics data can generally increase the performance of survival prognosis in breast cancer.

**Table 2:** Performances comparison with different combinations of multi-omics data by pairwise paired *t*-test, according to concordance index among 5-folds cross-validation results.

**Pairwise paired *T*-test**											
		**Set 2**									
		**ii**		**iii**		**iv**		**v**		**vi**	
		***t***	***P***	***t***	***P***	***t***	***P***	***t***	***P***	***t***	***P***
**Set 1**	**i**	−0.784	0.477	−0.676	0.536	−0.832	0.452	−2.928	0.043^*^	−3.315	0.030^*^
	**ii**	-	-	0.406	0.705	−0.487	0.652	−0.092	0.931	−0.652	0.550
	**iii**	–	–	–	–	0.247	0.817	−5.804	0.004^*^	−2.710	0.054
	**iv**	–	–	–	–	–	–	−4.168	0.014^*^	−3.603	0.023^*^
	**v**	–	–	–	–	–	–	–	–	−1.529	0.201

Note that a negative t-statistic indicated set 1 worse than set 2 in terms of performances. Multi-omics dataset applied as inputs: (i) mRNA-seq data (mRNA) (57 features); (ii) miRNA-seq data (miRNA) (12 features); (iii) integration of mRNA and miRNA (69 features); (iv) integration of mRNA, miRNA, copy number burden (CNB), and tumor mutation burden (TMB) (71 features); (v) integration of mRNA, miRNA, and demographical and clinical (diagnosis age, ER status, PR status) data (72 features); (vi) integration of mRNA, miRNA, CNB, TMB, and demographical and clinical (diagnosis age, ER status, PR status) data (74 features).

t-denotes the pairwise paired Student's t-test statistic, P denotes the p-value obtained. P-value < 0.05 are considered to be significant and indicated with * symbol.

Next, we compared SALMON to the state-of-the-art Deep Learning-based cancer survival prognosis model Cox-nnet (Ching et al., 2018), as well as another recently proposed DeepSurv (Katzman et al., 2018), and two traditional models generalized linear model with Cox regression (GLMNET) (Friedman et al., 2010) and RSF (Ishwaran et al., 2008). We further modified their original implementation with all omics data as inputs. As shown in Figure 2B, the median concordance index of SALMON (0.7285) was reported higher than the modified Cox-nnet (0.7234), DeepSurv (0.6563), GLMNET (0.6490), and RSF (0.6229). Compare to the modified Cox-nnet with similar performance in terms of concordance index, SALMON has a more significant result in log-rank test (*p*-value = 1.201E-04) than the modified Cox-nnet (*p*-value = 2.282E-04) with all testing sets and all 74 features as inputs (Figure S2). Between SALMON and the modified Cox-nnet the performance is insignificant (paired *t*-test statistic = −2.105, *p*-value = 0.103) suggesting these two methods are comparable. But from the neural network structure perspective, SALMON is more flexible since it separates forward propagation for each omics data, which enable a scalable integration of multi-omics data.

### Interpreting and Ranking the Importance of Co-expression Modules

Interpreting feature importance for neural networks has been studied over years. One way is to assign each feature be zero repeatedly, then the feature with lowest change of the resulting accuracy implies the least importance that affects to the prediction model. This approach is widely adopted for feature selection and ranking the importance of features in neural network (Setiono and Liu, 1997; Zhang, 2000; Sung and Mukkamala, 2003). Based on this approach, we analyzed the contribution of each eigengene's module to the final hazard ratio by forcing each input feature of the testing sets be zero. By feeding the modified testing sets to the pre-trained SALMON networks, we rank the importance of features by inspecting how much the concordance indices decreased. Features that decrease the testing concordance indices more are considered to be more important. At this moment, we integrated all omics data for training and testing. Table 3 presented top features that mostly reduced the concordance index. The leading two features are the diagnosis age and PR status, then five mRNA-seq co-expression modules are followed.

**Table 3:** Top features that reduced the concordance index, including two demographical and clinical features, and five mRNA-seq co-expression modules (eigengene matrices as inputs to the SALMON).

**Ranks**	**Feature names**	**Concordance index changed (median)**	**Highlighted genes/interpretations/enrichments or notes**
1	Diagnosis age	−0.1257	Age
2	PR status	−0.0343	Progesterone receptors status
3	Module 13	−0.0150	Genes MST1, CPT1B. CD8+, CD4+, Breast bulk tissue.
4	Module 47	−0.0071	Genes MAP3K7, CCNC. Cytoband chr6q14-q16 and chr6q21.
5	Module 5	−0.0059	Genes DDR2, FLNA, TCF4. Associated with extracellular matrix (ECM), cell adhesion, and cell migration.
6	Module 36	−0.0053	Gene SNW1. Cytoband chr14q23-q24 and chr14q31-q32.
7	Module 51	−0.0047	Genes TCP1, HDAC2. Cytoband chr6q14-q15and chr6q21-q26.

**Figure 3:**
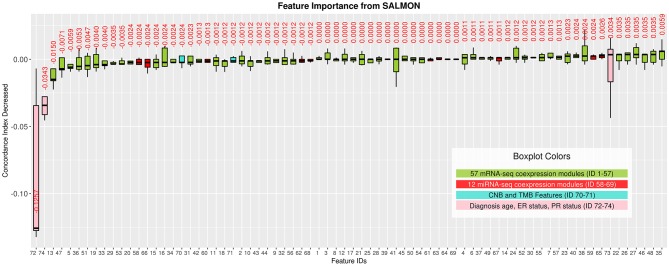
Features importance evaluated by the decrease of concordance index, sorted based on median values. Boxplots in Green: 57 mRNA co-expression module features (ID from 1 to 57); boxplots in red: 12 miRNA co-expression module features (ID from 58 to 69); boxplots in turquoise: copy number burden (CNB) and tumor mutation burden (TMB) features (ID from 70 to 71); boxplots in pink: demographical and clinical features (ID from 72 to 74).

Next, we selected those features (33 in total) of which their median values < 0 in Figure 3 and re-performed the training testing in SALMON. Results showed that before and after feature selection, the performances are insignificant in terms of concordance index (before feature selection: mean = 0.6918, median = 0.7285; after feature selection: mean = 0.7108, median = 0.7200; paired *t*-test statistic = −0.861, *p*-value = 0.438) (Figure S3). This implying that training with selected “important” multi-omics features instead of all can still preserve the prognosis performances.

### Identification of Breast Cancer Related Genes and Cytobands Associated With Important Modules

To inference the biological implication from the feature ranking, we performed Gene Ontology (GO) and cytoband enrichment from ToppGene Suite (https://toppgene.cchmc.org/) (Chen et al., 2009). Specifically, we focused on analyzing top five mRNA co-expression modules (Table 3). Totally we identified 10 genes such as MST1, CPT1B, MAP3K7, CCNC, etc. We also identified various enriched cytoband and other biological functions. Table 3 is further discussed and explained in Discussion section. Genes list within each mRNA-seq, miRNA-seq module is provided in Supplementary Material.

### Investigating Feature Importance With Different Age Groups

As shown in Figure 3, we observed the strong predictive power of diagnosis age, which is consistent with previous studies demonstrating age as one of the most prominent cancer risk factors (Adami et al., 1986). Thus, it is crucial to further investigate if patients in different groups can be stratified using the same set of features. In this paper, we define three age groups: (1) age in range 26–50 (191 patients), (2) age in range 51–70 (280 patients), (3) age in range 71–90 (112 patients) to represent younger, middle aged, and elderly patients. By training and testing these three distinct groups with SALMON algorithm, we aim to answer two questions: (1) whether the diagnosis age still be a strong factor that affect prognosis performance; (2) what are the differences of feature rankings between these three distinct groups.

**Figure 4:**
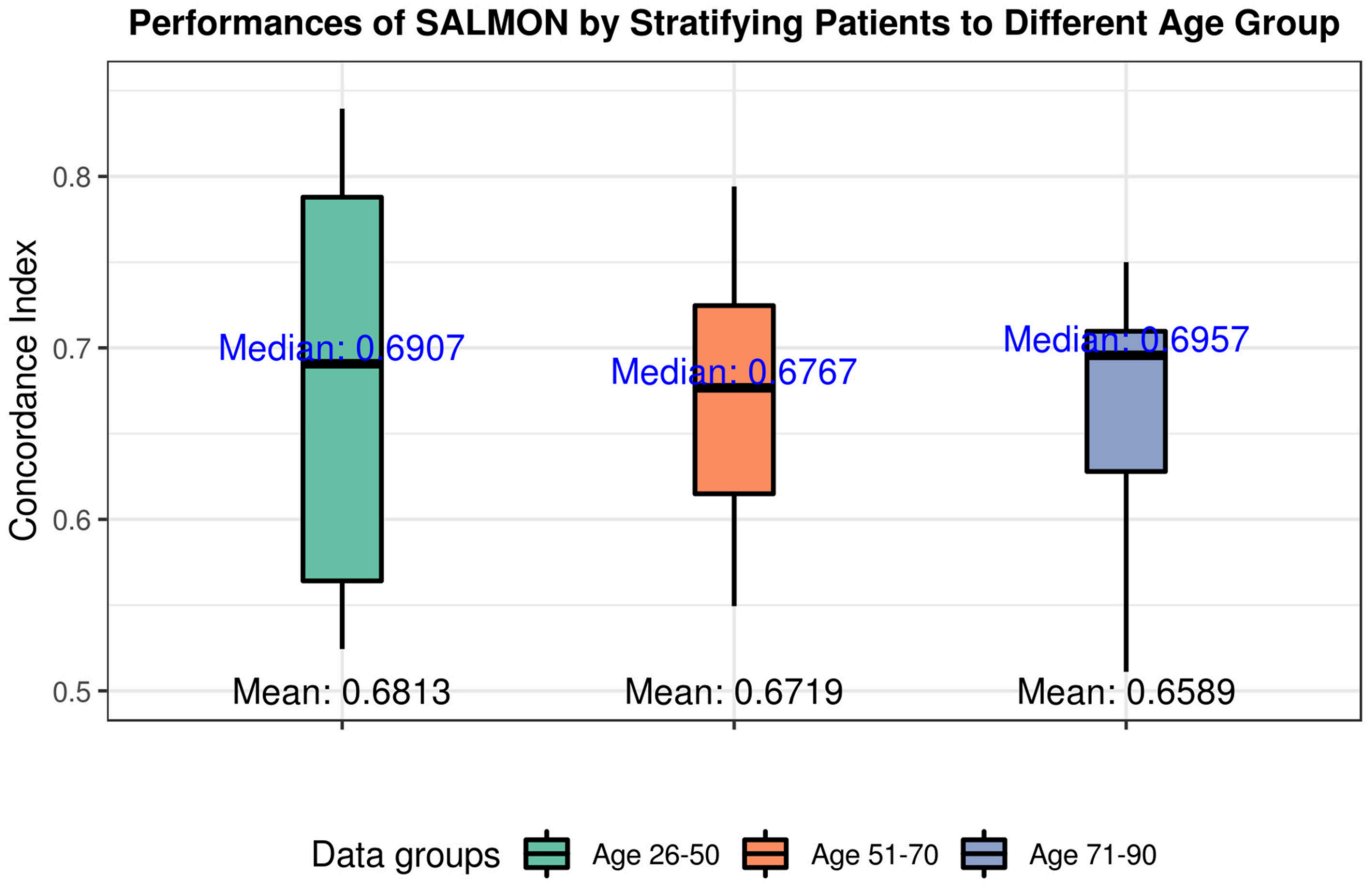
Performances of SALMON algorithm stratified by three age groups: 26–50 group; 51–70 group; 71–90 group with integrating all omics data (integration of mRNA, miRNA, CNB, TMB, diagnosis age, ER status, PR status).

The performances in terms of concordance index by integrating all omics and clinical data (including mRNA, miRNA, CNB, TMB, diagnosis age, ER status, PR status) are shown in Figure 4. As expected they are all slightly inferior than the performance when not stratifying patients by age (median = 0.7285; mean = 0.6918), there is not a statistical significant difference. When inspecting the feature rankings, as shown in Table 4, we observed that in the age group 26–50, PR status (Progesterone Receptors status) plays a pivotal role in prognosis, while other features do not have substantial contributions to the prognosis including the diagnosis age (we still listed some modules). This situation changed in the age group 51–70 as ER status (Estrogen Receptors status) becomes the most important feature, while diagnosis age ranked at #5 with only marginal contribution. In age group 71–90, neither ER, PR status nor diagnosis age ranked in the front, instead mRNA-seq co-expression modules appeared to have the major influence on prognosis. The top ranked modules are #11, #1, #29, #35, and #4. By performing enrichment analysis, we found that the module #11 is significantly enriched with epithelium development genes (GO:0060429, *p* = 2.253E-9); module #1 is significantly enriched with chromosome organization genes (GO:0051276, *p* = 5.344E-17) and two well-known breast cancer genes NCOA3 (Burwinkel et al., 2005) and FOXA1 (Meyer and Carroll, 2012; Rangel et al., 2018) were identified in module 1; module #29 was enriched on cytoband 19q13.41 (*p* = 1.517E-25) and are exclusively zinc-finger proteins; module #35 was enriched on cytoband 1q34 (*p* = 1.252E-15) and contains multiple genes which have been previously detected in multiple breast cancer studies including UQCRH, PSMB2, PPIH, and YBX1 (Miller et al., 2005; Pujana et al., 2007; Barry et al., 2010); and module #4 is highly enriched with mitotic cell cycle genes (GO:1903047, *p* = 2.183E-70) including well-known breast cancer genes such as MKI67 (Gyorffy et al., 2010) and AURKA (Cox et al., 2006). Detailed feature rankings are in Figures S5–S7.

**Table 4:** Top features that reduced the concordance indices.

**Ranks**	**Age group 26–50**		**Age group 51–70**		**Age group 71–90**	
	**Feature names**	**Concordance index changed (median)**	**Feature names**	**Concordance index changed (median)**	**Feature names**	**Concordance index changed (median)**
1	**PR status**	–**0.0247**	**ER status**	–**0.0807**	**Module 11**	–**0.0323**
2	Module 1	0	Module 13	−0.0221	**Module 1**	–**0.0233**
3	Module 2	0	Module 4	−0.0185	**Module 29**	–**0.0233**
4	Module 3	0	Module 5	−0.0150	**Module 35**	–**0.0233**
5	Module 4	0	Diagnosis age	−0.0150	**Module 4**	–**0.0222**

*Experiments performed separately with three age groups: 26–50 group; 51–70 group; 71–90 group, with integrating all omics data (integration of mRNA, miRNA, CNB, TMB, diagnosis age, ER status, PR status). Detailed feature rankings are in Figures S5–S7. The bold values are of our interests and are being discussed*.

## Discussion

In this work, we demonstrated the feasibility of breast cancer survival prognosis by integrating multi-omics data using Deep Learning-based approaches and opened up a new avenue for deriving new prognostic biomarkers in breast cancer. We introduced our SALMON (Survival Analysis Learning with Multi-Omics Neural Networks) algorithm with the implementation of Cox proportional hazards regression networks in breast invasive carcinoma. Instead of using gene level mRNA-seq or miRNA-seq data directly, SALMON adopts eigengene matrices as the network input derived from weighted gene co-expression network analysis. Unlike other algorithms, SALMON performs forward propagation separately with respect to each type of omics or clinical data in contrast with some other models such as Cox-nnet [which originally did not integrate multi-omics data nor use the co-expression modules as inputs (Ching et al., 2018)]. The separation of forward propagation prevents the interactions across omics data types thus enable easier examination of the module/feature importance for interpretability. It showed good prognosis results in terms of concordance index and log-rank test. Though experiments showed that SALMON has the competitive yet insignificantly superior performance compared to the state-of-the-art Cox-nnet (Ching et al., 2018), we have different paradigm in investigating how prognosis performance increases when integrating more omics and clinical data types, since other models such as Cox-nnet (Ching et al., 2018), DeepSurv (Katzman et al., 2018), etc. do not handle multi-omics data as input. The improved performances (concordance index) by integrating more omics data validates the hypothesis that integrative analysis enhances the survival prognosis accuracy for breast cancer. Moreover, using gene co-expression modules than gene expressions to reduce features upfront is the feature engineering technique we introduced based on bioinformatics techniques. By bridging the gap between gene co-expression analysis and Deep Learning, the advantages can be observed when we backtrack to identify the module/feature can affect the performances. The detected modules reveal clear cancer related biological processes, functions or structural variations allowing further biomedical investigations.

As feature importance has been conveyed and ranked from SALMON, we discovered that keeping only top important features can still preserve the testing performances. Based on features ranking, we also investigated the biological interpretation behind each demographical feature, clinical feature, and co-expression module. For the leading two features, since the importance of diagnosis age and PR status have been widely examined and recognized in breast cancer (Adami et al., 1986; Boyd et al., 1995; Huang et al., 2000; Bauer et al., 2007) and further confirmed by our results (Figure 2C), we focused on the top five mRNA-seq data co-expression modules ranked from 3 to 7. Those top five mRNA-seq data co-expression modules are: module #13, #47, #5, #36, #51.

In module #13, appears to be significantly associated with CD8+ T Cells (*p*-value = 6.54E-06) and CD4+ T Cells (*p*-value = 1.50E-02) based on Human Gene Atlas analysis. CD8+ and CD4+ T cells are important components of the immune system, which has been proved to have strong correlation with cancers (Hung et al., 1998; Hadrup et al., 2013). It contains multiple breast cancer related genes: (1) MST1 kinase, a core component of Hippo pathway, its phosphorylation can inhibit oncoproteins TAZ/YAP and regulate T-cell function. (Arash et al., 2017; Ercolani et al., 2017); (2) CPT1B, which encodes the critical enzyme for fatty acid beta-oxidation (FAO), the inhibition of FAO can inhibit breast cancer stem cells, chemoresistance, and breast tumor growth (Wang et al., 2018). In addition, tissues enrichment analysis using ARCHS4 (https://amp.pharm.mssm.edu/archs4/) also revealed that nearly one third of genes (11 out of 36) in this module were associated with breast cancer bulk tissue (*p*-value = 1.867E-03) (Figure S4).

In module #47, two genes are related to breast cancer have been identified: (1) MAP3K7, also known as TAK1, is a key mediator between survival and cell death in TNF-α-mediated signaling (Totzke et al., 2017); and (2) CCNC, an important transcriptional regulator whose higher expression is associated with shorter relapse-free survival (RFS) and impact the response to adjuvant therapy in breast cancer. Gene amplification of CCNC is also the most frequent type of genetic alterations in breast cancers (Broude et al., 2015). Module #47 was also enriched in cytoband chr6q.

In module #5, genes are highly enriched on tumor microenvironment (TME) related processes such as extracellular matrix (ECM), cell adhesion, and cell migration. Among them, DDR2 plays an indispensable role in a series of hypoxia-induced behaviors of breast cancer cells, such as migration, invasion, and epithelial-mesenchymal transition (EMT), the activated DDR2 can promote the metastasis of breast cancer (Ren et al., 2014). In addition, FLNA, whose overexpression is associated with the advanced stage, lymph node metastasis, and vascular or neural invasion of breast cancer (Feng et al., 2006). It also contributes the development of breast cancer (Tian et al., 2013). Finally, TCF4 is an important transcription factor, its loss is related with breast cancer chemoresistance (Ruiz de Garibay et al., 2018).

In module #36, SNW1 is a component of spliceosome in RNA splicing, its deletion can induce apoptosis, where the inhibition of SNW1 or its associating proteins may be a novel therapeutic strategy for cancer treatment (Sato et al., 2015). Module #36 was also enriched in cytoband chr14q23-q24 and chr14q31-q32.

In module #51, TCP1 functioned as a cytosolic chaperone in the biogenesis of tubulin (Yaffe et al., 1992), which has been proved to have an association with breast cancer (Bassiouni et al., 2016). HDAC2, its overexpression has a correlation with DNA-damage response and promote tumor progression (Shan et al., 2017). Module #51 was also enriched on cytoband chr6q.

Instead of identified breast cancer related genes, the Enrichment analysis in selected modules also revealed important biological functions. Module 47 and 51 were enriched in chr6q. Not surprisingly, previous studies have identified the frequent alterations at chr6q in archival breast cancer specimens (Shadeo and Lam, 2006), while chr6q21 is hotspots copy number alteration region (Chin et al., 2007). The copy number alterations at chr6q26 can affect MAP3K4, plays an important role of epidermal growth factor receptor pathway (Shadeo and Lam, 2006). Module 36 was enriched in chr14q, the cytoband where the high-level alterations at 14q31.3-32.12 were found in breast cancer from Shadeo and Lam (2006). Besides, the deletion of chr14q is a common feature of tumors with BRCA2 mutations (Rouault et al., 2012). Modules 5 was specifically associated with TME related biological process such as extracellular matrix (ECM), cell adhesion and cell migration. All these GO Biological Processes (BPs) have been shown to play pivotal roles in TME development in cancers while TME has now been widely recognized as a critical participant in tumor progression (Quail and Joyce, 2013). Abnormal ECM in tumors can promote the aggressiveness of breast cancer (Robertson, 2016). Cell adhesion as a common event in cancer can promote cell growth as well as tumor dissemination (Moh and Shen, 2009; Saadatmand et al., 2013). All these discoveries not only confirmed the existed literatures for breast cancer, but also justified the feature importance that SALMON generated.

Another interesting finding is that no miRNA-seq module was ranked in top features although miRNA-seq modules show a better prognosis performance than mRNA-seq modules. This could due to the modules within miRNA-seq are more dependent with each other than the modules within mRNA-seq, thus simply knock out one module/feature may not reduce the performance too much. Indeed, by performing pair-wised Pearson correlation analysis, we found 3.03% miRNA-seq modules has strong correlations (Pearson ρ>0.8), while in mRNA-seq modules this ratio is down to 0.94%. It leads us a new perspective to inspect modules dependency in the future.

Since we confirmed that diagnosis age is the most powerful predictor, we examined the feature rankings with three different age groups, namely, younger group (age 26–50), middle aged group (age 51–70), and elderly group (age 71–90). We confirmed that by separating the 583 patients to three distinct age groups, the diagnosis age becomes unimportant to the prognosis outcome. While in younger group, PR status is the most important feature. In middle aged group, ER status is the most important feature. When we inspected the elderly group with age in range 71–90, we found that only mRNA-seq co-expression modules were ranked at the top and the five most conspicuous ones are modules #11, #1, #29, #35, and #4. These observations suggest that specific biological processes may play different roles in breast cancer patients of different ages while different biomarkers and predictive models may be needed for different age groups. Further inspection of the modules found that three of these modules are related to known breast cancer related processes such as epithelium development (Vincent-Salomon and Thiery, 2003), chromosome organization (Muleris et al., 1995), and mitotic cell cycle (Kastan and Bartek, 2004) including well-known breast cancers genes such as NCOA3, AURKA, MKI67, and FOXA1. The other two modules are highly enriched on specific cytobands on different chromosomes, implying potential copy number variations on these regions. Indeed, both cytobands (19q13.41 and 1q34) are known to be associated with breast cancer outcomes (Han et al., 2006; Ton et al., 2009). For module #35, while most of the genes locate on 1q34, many of the genes such as UQCRH, PSMB2, PPIH, and YBX1 are involved in RNA processing and have been identified with breast cancer in multiple studies (Miller et al., 2005; Pujana et al., 2007; Barry et al., 2010). Interestingly, all genes identified from module #29 are zinc finger transcription factors. While it is not clear if any of them are specifically related to breast cancer, it is of great interest to further investigate the roles of the ZNF family genes in breast cancer development.

## Conclusion

We performed survival prognosis on breast cancer, proposed a Deep Learning-based algorithm SALMON (Survival Analysis Learning with Multi-Omics Network) by integrating Cox proportional hazards model and adopting gene co-expression network analysis results as input, and predict patient hazard ratios precisely. Performances (concordance index and log-rank test *p*-value) improved when more omics data integrated to the input of SALMON. SALMON also showed a competitive performance compared to other Deep Learning survival prognosis model. By inspecting how each feature contributes to the hazard ratios, SALMON confirmed certain mRNA-seq co-expression modules and clinical information, which play pivotal roles in breast cancer prognosis, revealed several biological functions. By further stratifying patients with diagnosis age, SALMON confirmed that different age groups have different main features that controls survival prognosis performance. To sum up, SALMON fuses the gene co-expression network analysis, Deep Learning technique, feature selection, Cox proportional hazard model, integrative analysis, and module-level enrichment analysis altogether, offers a new avenue for the future integrative analysis and Deep Learning-based cancer survival prognosis.

## Data Availability

All datasets generated for this study are included in the manuscript and/or the supplementary files.

## Author Contributions

ZHu conceived and designed the algorithm and analysis, conducted the experiments, and wrote the paper. XZ, KH, ZHu performed the biological analysis and wrote the paper. SX, JZ performed the biological analysis. TJ, CY, ZHa collected the data. TJ, BH, KH edited the paper. JZ, PS, MR, ZHa, KH provided the research guide. PS, ZHa, KH supervised this project.

### Conflict of Interest Statement

The authors declare that the research was conducted in the absence of any commercial or financial relationships that could be construed as a potential conflict of interest.

## References

[B1] Adami H. O. Malker B. Holmberg L. Persson I. Stone B. (1986). The relation between survival and age at diagnosis in breast cancer. N. Engl. J. Med. 315, 559–563. 10.1056/NEJM198608283150906 3736639

[B2] Arash E. H. Shiban A. Song S. Y. Attisano L. (2017). MARK4 inhibits Hippo signaling to promote proliferation and migration of breast cancer cells. EMBO Rep. 18, 420–436. 10.15252/embr.201642455 28183853 PMC5331264

[B3] Barry W. T. Kernagis D. N. Dressman H. K. Griffis R. J. Hunter J. D. Olson J. A.. (2010). Intratumor heterogeneity and precision of microarray-based predictors of breast cancer biology and clinical outcome. J. Clin. Oncol. 28, 2198–2206. 10.1200/JCO.2009.26.7245 20368555 PMC2860437

[B4] Bassiouni R. Nemec K. N. Iketani A. Flores O. Showalter A. Khaled A. S.. (2016). Chaperonin containing TCP-1 protein level in breast cancer cells predicts therapeutic application of a cytotoxic peptide. Clin. Cancer Res. 22, 4366–4379. 10.1158/1078-0432.CCR-15-2502 27012814 PMC5010522

[B5] Bauer K. R. Brown M. Cress R. D. Parise C. A. Caggiano V. (2007). Descriptive analysis of estrogen receptor (ER)negative, progesterone receptor (PR)-negative, and HER2-negative invasive breast cancer, the so-called triple-negative phenotype - a population-based study from the California Cancer Registry. Cancer 109, 1721–1728. 10.1002/cncr.22618 17387718

[B6] Birkbak N. J. Kochupurakkal B. Izarzugaza J. M. Eklund A. C. Li Y. Liu J. (2013). Tumor mutation burden forecasts outcome in ovarian cancer with BRCA1 or BRCA2 mutations. PLoS ONE 8:e80023 10.1371/journal.pone.0080023 24265793 PMC3827141

[B7] Boyd N. F. Byng J. W. Jong R. A. Fishell E. K. Little L. E. Miller A. B.. (1995). Quantitative classification of mammographic densities and breast cancer risk: results from the Canadian National Breast Screening Study. J. Natl. Cancer Inst. 87, 670–675. 10.1093/jnci/87.9.670 7752271

[B8] Bradley A. P. (1997). The use of the area under the roc curve in the evaluation of machine learning algorithms. Pattern Recognit. 30, 1145–1159. 10.1016/S0031-3203(96)00142-2

[B9] Broude E. V. Gyorffy B. Chumanevich A. A. Chen M. Q. McDermott M. S. J. Shtutman M.. (2015). Expression of CDK8 and CDK8-interacting genes as potential biomarkers in breast cancer. Curr. Cancer Drug Targets 15, 739–749. 10.2174/156800961508151001105814 26452386 PMC4755306

[B10] Burwinkel B. Wirtenberger M. Klaes R. Schmutzler R. K. Grzybowska E. Forsti A.. (2005). Association of NCOA3 polymorphisms with breast cancer risk. Clin. Cancer Res. 11, 2169–2174. 10.1158/1078-0432.CCR-04-1621 15788663

[B11] Caruana R. Lawrence S. Giles L. (2001). Overfitting in neural nets: backpropagation, conjugate gradient, and early stopping, in Advances in Neural Information Processing Systems 13, Papers from Neural Information Processing Systems (NIPS) 2000 (Denver, CO), 402–408.

[B12] Chalmers Z. R. Connelly C. F. Fabrizio D. Gay L. Ali S. M. Ennis R.. (2017). Analysis of 100,000 human cancer genomes reveals the landscape of tumor mutational burden. Genome Med. 9:34. 10.1186/s13073-017-0424-2 28420421 PMC5395719

[B13] Chandran V. Coppola G. Nawabi H. Omura T. Versano R. Huebner E. A.. (2016). A systems-level analysis of the peripheral nerve intrinsic axonal growth program. Neuron 89, 956–970. 10.1016/j.neuron.2016.01.034 26898779 PMC4790095

[B14] Chaudhary K. Poirion O. B. Lu L. Garmire L. X. (2018). Deep learning-based multi-omics integration robustly predicts survival in liver cancer. Clin. Cancer Res. 24, 1248–1259. 10.1158/1078-0432.CCR-17-0853 28982688 PMC6050171

[B15] Chen J. Bardes E. E. Aronow B. J. Jegga A. G. (2009). ToppGene suite for gene list enrichment analysis and candidate gene prioritization. Nucleic Acids Res. 37, W305–W311. 10.1093/nar/gkp427 19465376 PMC2703978

[B16] Cheng J. Zhang J. Han Y. Wang X. Ye X. Meng Y.. (2017). Integrative analysis of histopathological images and genomic data predicts clear cell renal cell carcinoma prognosis. Cancer Res. 77, e91–e100. 10.1158/0008-5472.CAN-17-0313 29092949 PMC7262576

[B17] Chin S. F. Teschendorff A. E. Marioni J. C. Wang Y. Barbosa-Morais N. L. Thorne N. P.. (2007). High-resolution aCGH and expression profiling identifies a novel genomic subtype of ER negative breast cancer. Genome Biol. 8:R215. 10.1186/gb-2007-8-10-r215 17925008 PMC2246289

[B18] Ching T. Zhu X. Garmire L. X. (2018). Cox-nnet: an artificial neural network method for prognosis prediction of high-throughput omics data. PLoS Comput. Biol. 14:e1006076. 10.1371/journal.pcbi.1006076 29634719 PMC5909924

[B19] Cox D. G. Hankinson S. E. Hunter D. J. (2006). Polymorphisms of the AURKA (STK15/Aurora Kinase) gene and breast cancer risk (United States). Cancer Causes Control 17, 81–83. 10.1007/s10552-005-0429-9 16411056

[B20] Ercolani C. Di Benedetto A. Terrenato I. Pizzuti L. Di Lauro L. Sergi D.. (2017). Expression of phosphorylated Hippo pathway kinases (MST1/2 and LATS1/2) in HER2-positive and triple-negative breast cancer patients treated with neoadjuvant therapy. Cancer Biol. Ther. 18, 339–346. 10.1080/15384047.2017.1312230 28387539 PMC5499753

[B21] Feng Y. Chen M. H. Moskowitz I. P. Mendonza A. M. Vidali L. Nakamura F.. (2006). Filamin A (FLNA) is required for cell-cell contact in vascular development and cardiac morphogenesis. Proc. Natl. Acad. Sci. U.S.A. 103, 19836–19841. 10.1073/pnas.0609628104 17172441 PMC1702530

[B22] Friedman J. Hastie T. Tibshirani R. (2010). Regularization paths for generalized linear models via coordinate descent. J. Stat. Softw. 33, 1–22. 10.18637/jss.v033.i01 20808728 PMC2929880

[B23] Geman S. Bienenstock E. Doursat R. (1992). Neural networks and the bias variance dilemma. Neural Comput. 4, 1–58. 10.1162/neco.1992.4.1.1

[B24] Golub G. H. Reinsch C. (1970). Singular value decomposition and least squares solutions. Numerische Math. 14, 403–420. 10.1007/BF02163027

[B25] Goodman A. M. Kato S. Bazhenova L. Patel S. P. Frampton G. M. Miller V.. (2017). Tumor mutational burden as an independent predictor of response to immunotherapy in diverse cancers. Mol. Cancer Ther. 16, 2598–2608. 10.1158/1535-7163.MCT-17-0386 28835386 PMC5670009

[B26] Gupta A. Mutebi M. Bardia A. (2015). Gene-expression-based predictors for breast cancer. Ann. Surg. Oncol. 22, 3418–3432. 10.1245/s10434-015-4703-0 26215189

[B27] Gyorffy B. Lanczky A. Eklund A. C. Denkert C. Budczies J. Li Q.. (2010). An online survival analysis tool to rapidly assess the effect of 22,277 genes on breast cancer prognosis using microarray data of 1,809 patients. Breast Cancer Res. Treat. 123, 725–731. 10.1007/s10549-009-0674-9 20020197

[B28] Hadrup S. Donia M. Thor Straten P. (2013). Effector CD4 and CD8 T cells and their role in the tumor microenvironment. Cancer Microenviron. 6, 123–133. 10.1007/s12307-012-0127-6 23242673 PMC3717059

[B29] Han W. Han M. R. Kang J. J. Bae J. Y. Lee J. H. Bae Y. J.. (2006). Genomic alterations identified by array comparative genomic hybridization as prognostic markers in tamoxifen-treated estrogen receptor-positive breast cancer. BMC Cancer 6:92. 10.1186/1471-2407-6-92 16608533 PMC1459182

[B30] Han Z. Johnson T. Zhang J. Zhang X. Huang K. (2017). Functional virtual flow cytometry: a visual analytic approach for characterizing single-cell gene expression patterns. Biomed. Res. Int. 2017:3035481 10.1155/2017/303548 28798928 PMC5536134

[B31] Han Z. Zhang J. Sun G. Liu G. Huang K. (2016). A matrix rank based concordance index for evaluating and detecting conditional specific co-expressed gene modules. BMC Genomics 17(Suppl. 7):519. 10.1186/s12864-016-2912-y 27556416 PMC5001231

[B32] Horvath S. Zhang Y. Langfelder P. Kahn R. S. Boks M. P. van Eijk K.. (2012). Aging effects on DNA methylation modules in human brain and blood tissue. Genome Biol. 13:R97. 10.1186/gb-2012-13-10-r97 23034122 PMC4053733

[B33] Huang S. Chaudhary K. Garmire L. X. (2017). More is better: recent progress in multi-omics data integration methods. Front. Genet. 8:84. 10.3389/fgene.2017.00084 28670325 PMC5472696

[B34] Huang W. Y. Newman B. Millikan R. C. Schell M. J. Hulka B. S. Moorman P. G. (2000). Hormone-related factors and risk of breast cancer in relation to estrogen receptor and progesterone receptor status. Am. J. Epidemiol. 151, 703–714. 10.1093/oxfordjournals.aje.a010265 10752798

[B35] Hung K. Hayashi R. Lafond-Walker A. Lowenstein C. Pardoll D. Levitsky H. (1998). The central role of CD4(+) T cells in the antitumor immune response. J. Exp. Med. 188, 2357–2368. 10.1084/jem.188.12.2357 9858522 PMC2212434

[B36] Ishwaran H. Kogalur U. B. Blackstone E. H. Lauer M. S. (2008). Random survival forests. Ann. Appl. Stat. 2, 841–860. 10.1214/08-AOAS169

[B37] Kastan M. B. Bartek J. (2004). Cell-cycle checkpoints and cancer. Nature 432, 316–323. 10.1038/nature03097 15549093

[B38] Katzman J. L. Shaham U. Cloninger A. Bates J. Jiang T. T. Kluger Y. (2018). DeepSurv: personalized treatment recommender system using a Cox proportional hazards deep neural network. BMC Med. Res. Methodol. 18:24. 10.1186/s12874-018-0482-1 29482517 PMC5828433

[B39] Kingma D. Ba J. (2015). Adam: a method for stochastic optimization, in Proceedings of the 3rd International Conference on Learning Representations (ICLR 2015).

[B40] Kuleshov M. V. Jones M. R. Rouillard A. D. Fernandez N. F. Duan Q. Wang Z.. (2016). Enrichr: a comprehensive gene set enrichment analysis web server 2016 update. Nucleic Acids Res. 44, W90–W97. 10.1093/nar/gkw377 27141961 PMC4987924

[B41] Langfelder P. Horvath S. (2008). WGCNA: an R package for weighted correlation network analysis. BMC Bioinformatics 9:559. 10.1186/1471-2105-9-559 19114008 PMC2631488

[B42] Liu G. Dong C. Liu L. (2016). Integrated multiple “-omics” data reveal subtypes of hepatocellular carcinoma. PLoS ONE 11:e0165457. 10.1371/journal.pone.0165457 27806083 PMC5091875

[B43] Ma T. Zhang A. (2018). Multi-view factorization AutoEncoder with network constraints for multi-omic integrative analysis, in IEEE International Conference on Bioinformatics and Biomedicine (BIBM) (IEEE). 10.1109/BIBM.2018.8621379

[B44] Mantel N. (1966). Evaluation of survival data and two new rank order statistics arising in its consideration. Cancer Chemother. Rep. 50, 163–170. 5910392

[B45] Marshall C. R. Howrigan D. P. Merico D. Thiruvahindrapuram B. Wu W. Greer D. S.. (2017). Contribution of copy number variants to schizophrenia from a genome-wide study of 41,321 subjects. Nat. Genet. 49, 27–35. 10.1038/ng.3725 27869829 PMC5737772

[B46] Meyer K. B. Carroll J. S. (2012). FOXA1 and breast cancer risk. Nat. Genet. 44, 1176–1177. 10.1038/ng.2449 23104063

[B47] Miller L. D. Smeds J. George J. Vega V. B. Vergara L. Ploner A.. (2005). An expression signature for p53 status in human breast cancer predicts mutation status, transcriptional effects, and patient survival. Proc. Natl. Acad. Sci. U.S.A. 102, 13550–13555. 10.1073/pnas.0506230102 16141321 PMC1197273

[B48] Moh M. C. Shen S. L. (2009). The roles of cell adhesion molecules in tumor suppression and cell migration a new paradox. Cell Adh. Migr. 3, 334–336. 10.4161/cam.3.4.9246 19949308 PMC2802741

[B49] Mukaka M. M. (2012). Statistics corner: a guide to appropriate use of correlation coefficient in medical research. Malawi Med. J. 24, 69–71. 23638278 PMC3576830

[B50] Muleris M. Almeida A. Gerbault-Seureau M. Malfoy B. Dutrillaux B. (1995). Identification of amplified DNA sequences in breast cancer and their organization within homogeneously staining regions. Genes Chromosomes Cancer 14, 155–163. 10.1002/gcc.2870140302 8589031

[B51] Nagini S. (2017). Breast cancer: current molecular therapeutic targets and new players. Anticancer. Agents Med. Chem. 17, 152–163. 10.2174/1871520616666160502122724 27137076

[B52] Nassar F. J. Nasr R. Talhouk R. (2017). MicroRNAs as biomarkers for early breast cancer diagnosis, prognosis and therapy prediction. Pharmacol. Ther. 172, 34–49. 10.1016/j.pharmthera.2016.11.012 27916656

[B53] Ng A. Y. Jordan M. I. Weiss Y. (2002). On spectral clustering: analysis and an algorithm, in Advances in Neural Information Processing Systems (MIT Press), 849–856.

[B54] Pereira B. Chin S. F. Rueda O. M. Vollan H. K. Provenzano E. Bardwell H. A.. (2016). The somatic mutation profiles of 2,433 breast cancers refines their genomic and transcriptomic landscapes. Nat. Commun. 7:11479. 10.1038/ncomms11479 27161491 PMC4866047

[B55] Poirion O. B. Chaudhary K. Garmire L. X. (2018). Deep learning data integration for better risk stratification models of bladder cancer. AMIA Jt. Summits Transl. Sci. Proc. 2017, 197–206. 29888072 PMC5961799

[B56] Pujana M. A. Han J. D. Starita L. M. Stevens K. N. Tewari M. Ahn J. S.. (2007). Network modeling links breast cancer susceptibility and centrosome dysfunction. Nat. Genet. 39, 1338–1349. 10.1038/ng.2007.2 17922014

[B57] Quail D. F. Joyce J. A. (2013). Microenvironmental regulation of tumor progression and metastasis. Nat. Med. 19, 1423–1437. 10.1038/nm.3394 24202395 PMC3954707

[B58] Ramazzotti D. Lal A. Wang B. Batzoglou S. Sidow A. (2018). Multi-omic tumor data reveal diversity of molecular mechanisms that correlate with survival. Nat. Commun. 9:4453. 10.1038/s41467-018-06921-8 30367051 PMC6203719

[B59] Rangel N. Fortunati N. Osella-Abate S. Annaratone L. Isella C. Catalano M. G.. (2018). FOXA1 and AR in invasive breast cancer: new findings on their co-expression and impact on prognosis in ER-positive patients. BMC Cancer 18:703. 10.1186/s12885-018-4624-y 29970021 PMC6029370

[B60] Ren T. Zhang W. Liu X. Zhao H. Zhang J. Zhang J.. (2014). Discoidin domain receptor 2 (DDR2) promotes breast cancer cell metastasis and the mechanism implicates epithelial-mesenchymal transition programme under hypoxia. J. Pathol. 234, 526–537. 10.1002/path.4415 25130389

[B61] Robertson C. (2016). The extracellular matrix in breast cancer predicts prognosis through composition, splicing, and crosslinking. Exp. Cell Res. 343, 73–81. 10.1016/j.yexcr.2015.11.009 26597760

[B62] Rouault A. Banneau G. MacGrogan G. Jones N. Elarouci N. Barouk-Simonet E.. (2012). Deletion of chromosomes 13q and 14q is a common feature of tumors with BRCA2 mutations. PLoS ONE 7:e52079. 10.1371/journal.pone.0052079 23284877 PMC3528765

[B63] Ruiz de Garibay G. Mateo F. Stradella A. Valdes-Mas R. Palomero L. Serra-Musach J.. (2018). Tumor xenograft modeling identifies an association between TCF4 loss and breast cancer chemoresistance. Dis. Model. Mech. 11:dmm032292. 10.1242/dmm.032292 29666142 PMC5992609

[B64] Saadatmand S. de Kruijf E. M. Sajet A. Dekker-Ensink N. G. van Nes J. G. H. Putter H.. (2013). Expression of cell adhesion molecules and prognosis in breast cancer. Br. J. Surg. 100, 252–260. 10.1002/bjs.8980 23175431

[B65] Santosa F. Symes W. W. (1986). Linear inversion of band-limited reflection seismograms. SIAM J. Sci. Stat. Comput. 7, 1307–1330. 10.1137/0907087

[B66] Sato N. Maeda M. Sugiyama M. Ito S. Hyodo T. Masuda A.. (2015). Inhibition of SNW1 association with spliceosomal proteins promotes apoptosis in breast cancer cells. Cancer Med. 4, 268–277. 10.1002/cam4.366 25450007 PMC4329010

[B67] Schmidhuber J. (2015). Deep learning in neural networks: an overview. Neural Netw. 61, 85–117. 10.1016/j.neunet.2014.09.003 25462637

[B68] Setiono R. Liu H. (1997). Neural-network feature selector. IEEE Trans. Neural Netw. 8, 654–662. 10.1109/72.572104 18255668

[B69] Shadeo A. Lam W. L. (2006). Comprehensive copy number profiles of breast cancer cell model genomes. Breast Cancer Res. 8:R9. 10.1186/bcr1370 16417655 PMC1413994

[B70] Shan W. Jiang Y. Yu H. Huang Q. Liu L. Guo X.. (2017). HDAC2 overexpression correlates with aggressive clinicopathological features and DNA-damage response pathway of breast cancer. Am. J. Cancer Res. 7, 1213–1226. 28560068 PMC5446485

[B71] Shao W. Cheng J. Sun L. Han Z. Feng Q. Zhang D. (2018). Ordinal multi-modal feature selection for survival analysis of early-stage renal cancer, in International Conference on Medical Image Computing and Computer-Assisted Intervention (Granada: Springer), 648–656.

[B72] Shroff S. Zhang J. Huang K. (2016). Gene co-expression analysis predicts genetic variants associated with drug responsiveness in lung cancer. AMIA Jt Summits Transl. Sci. Proc. 2016, 32–41. 27570645 PMC5001757

[B73] Steck H. Krishnapuram B. Dehing-oberije C. Lambin P. Raykar V. C. (2007). On ranking in survival analysis: bounds on the concordance index, in Advances in Neural Information Processing Systems (Vancouver, BC), 1209–1216.

[B74] Sun D. Wang M. Li A. (2018). A multimodal deep neural network for human breast cancer prognosis prediction by integrating multi-dimensional data. IEEE/ACM Trans. Comput. Biol. Bioinform. 99:1 10.1109/TCBB.2018.2806438 29994639

[B75] Sung A. H. Mukkamala S. (2003). Identifying important features for intrusion detection using support vector machines and neural networks, in 2003 Symposium on Applications and the Internet, Proceedings (Orlando, FL), 209–216. 10.1109/SAINT.2003.1183050

[B76] Thomas A. Routh E. D. Pullikuth A. Jin G. Su J. Chou J. W.. (2018). Tumor mutational burden is a determinant of immune-mediated survival in breast cancer. Oncoimmunology 7:e1490854. 10.1080/2162402X.2018.1490854 30386679 PMC6207420

[B77] Tian H. M. Liu X. H. Han W. Zhao L. L. Yuan B. Yuan C. J. (2013). Differential expression of filamin A and its clinical significance in breast cancer. Oncol. Lett. 6, 681–686. 10.3892/ol.2013.1454 24137390 PMC3789035

[B78] Ton C. Guenthoer J. Porter P. L. (2009). Somatic alterations and implications in breast cancer, in Role of Genetics in Breast and Productive Cancers, ed Welcsh P. (Seattle, WA: Springer), 183–213. 10.1007/978-1-4419-0477-5_9

[B79] Totzke J. Gurbani D. Raphemot R. Hughes P. F. Bodoor K. Carlson D. A.. (2017). Takinib, a selective TAK1 inhibitor, broadens the therapeutic efficacy of TNF-alpha inhibition for cancer and autoimmune disease. Cell Chem. Biol. 24, 1029–1039 e1027. 10.1016/j.chembiol.2017.07.011 28820959 PMC5576570

[B80] Vincent-Salomon A. Thiery J. P. (2003). Host microenvironment in breast cancer development: epithelial-mesenchymal transition in breast cancer development. Breast Cancer Res. 5, 101–106. 10.1186/bcr578 12631389 PMC154156

[B81] Wang T. Fahrmann J. F. Lee H. Li Y. J. Tripathi S. C. Yue C. (2018). JAK/STAT3-regulated fatty acid beta-oxidation is critical for breast cancer stem cell self-renewal and chemoresistance. Cell Metab. 27, 136–150 e135. 10.1016/j.cmet.2018.04.018 29249690 PMC5777338

[B82] Weigend A. S. Rumelhart D. E. Huberman B. A. (1990). Generalization by weight-elimination with application to forecasting, in Advances in Neural Information Processing Systems (Denver, CO), 875–882

[B83] Wu X. F. Ye Y. Q. Barcenas C. H. Chow W. H. Meng Q. H. Chavez-MacGregor M.. (2017). Personalized prognostic prediction models for breast cancer recurrence and survival incorporating multidimensional data. J. Natl. Cancer Inst. 109. 10.1093/jnci/djw314 28376179 PMC6279311

[B84] Xiang S. Huang Z. Wang T. Han Z. Yu C. Y. Ni D.. (2018). Condition-specific gene co-expression network mining identifies key pathways and regulators in the brain tissue of Alzheimer's disease patients. 11:115. 10.1186/s12920-018-0431-1 30598117 PMC6311927

[B85] Xiang Y. Zhang C. Q. Huang K. (2012). Predicting glioblastoma prognosis networks using weighted gene co-expression network analysis on TCGA data. BMC Bioinformatics 13:S12. 10.1186/1471-2105-13-S2-S12 22536863 PMC3305748

[B86] Yaffe M. B. Farr G. W. Miklos D. Horwich A. L. Sternlicht M. L. Sternlicht H. (1992). TCP1 complex is a molecular chaperone in tubulin biogenesis. Nature 358, 245–248. 10.1038/358245a0 1630491

[B87] Zhang G. Q. P. (2000). Neural networks for classification: a survey. IEEE Trans. Syst. Man Cybern. Part C Appl. Rev. 30, 451–462. 10.1109/5326.897072

[B88] Zhang J. Abrams Z. Parvin J. D. Huang K. (2016). Integrative analysis of somatic mutations and transcriptomic data to functionally stratify breast cancer patients. BMC Genomics 17(Suppl. 7):513. 10.1186/s12864-016-2902-0 27556157 PMC5001235

[B89] Zhang J. Huang K. (2014). Normalized lmQCM: an algorithm for detecting weak quasi-cliques in weighted graph with applications in gene co-expression module discovery in cancers. Cancer Inform. 13(Suppl. 3), 137–146. 10.4137/CIN.S14021 27486298 PMC4962959

[B90] Zhang J. Huang K. (2017). Pan-cancer analysis of frequent DNA co-methylation patterns reveals consistent epigenetic landscape changes in multiple cancers. BMC Genomics 18(Suppl. 1):1045. 10.1186/s12864-016-3259-0 28198667 PMC5310283

[B91] Zhang J. Lu K. W. Xiang Y. Islam M. Kotian S. Kais Z.. (2012). Weighted frequent gene co-expression network mining to identify genes involved in genome stability. PLoS Comput. Biol. 8:e1002656. 10.1371/journal.pcbi.1002656 22956898 PMC3431293

[B92] Zhang J. Ni S. Xiang Y. Parvin J. D. Yang Y. Zhou Y.. (2013). Gene co-expression analysis predicts genetic aberration loci associated with colon cancer metastasis. Int. J. Comput. Biol. Drug Des. 6, 60–71. 10.1504/IJCBDD.2013.052202 23428474

[B93] Zhang L. Lv C. Jin Y. Cheng G. Fu Y. Yuan D.. (2018a). Deep learning-based multi-omics data integration reveals two prognostic subtypes in high-risk neuroblastoma. Front. Genet. 9:477. 10.3389/fgene.2018.00477 30405689 PMC6201709

[B94] Zhang Z. Zhao Y. Liao X. Shi W. Li K. Zou Q. (2018b). Deep learning in omics: a survey and guideline. Brief. Funct. Genomics. 18, 41–57. 10.1093/bfgp/ely030 30265280

