# Supplementary material for: SALMON: Survival Analysis Learning With Multi-Omics Neural Networks on Breast Cancer
Source: Front Genet. 2019 Mar 8;10:166. doi: 10.3389/fgene.2019.00166 (PMC6419526; doi:10.3389/fgene.2019.00166)
Supplement: Supplementary file 1 [file Table_1.docx]

SALMON: Survival Analysis Learning with Multi-Omics Neural Networks on Breast Cancer

Supplementary Material


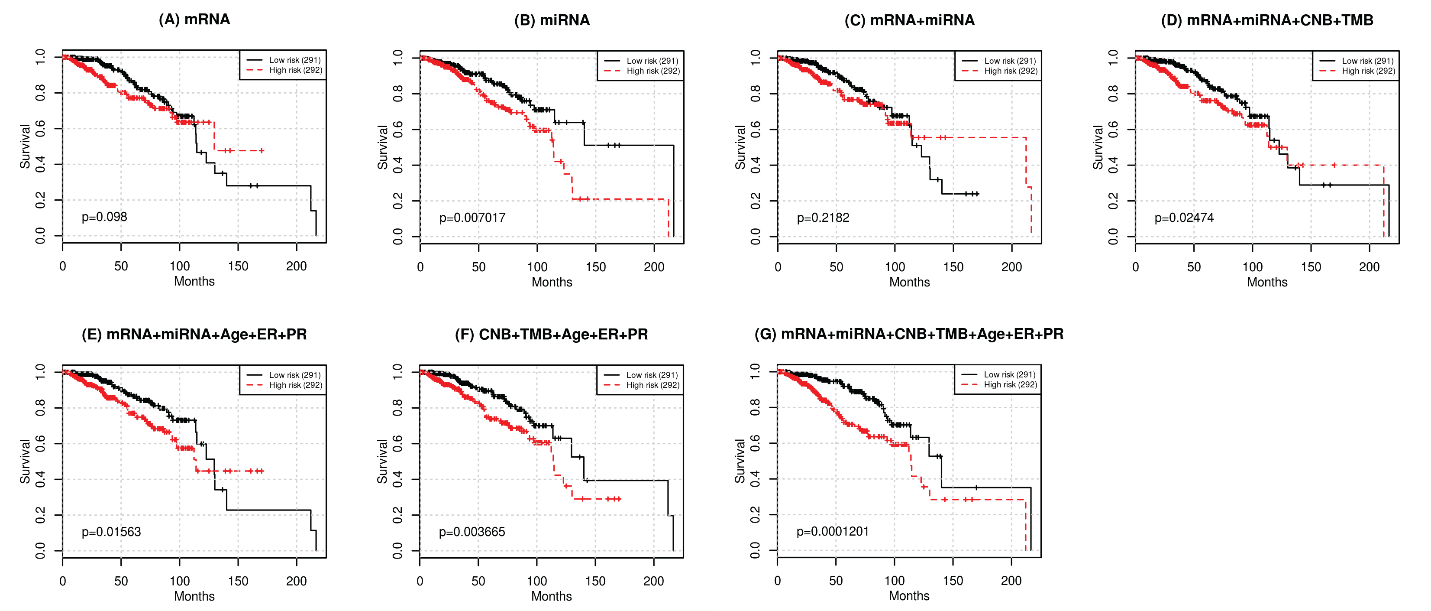


Figure S1. Kaplan-Meier plot of survival prognosis. Hazard ratios were derived from all five testing sets. Log-rank test was used to find the corresponding p-value with low risk and high risk groups dichotomized by the median hazard ratio. Omics data used for training and testing: (A) mRNA-seq data (mRNA) (57 features); (B) miRNA-seq data (miRNA) (12 features); (C) integration of mRNA and miRNA (69 features); (D) integration of mRNA, miRNA, copy number burden (CNB), and tumor mutation burden (TMB) (71 features); (E) integration of mRNA, miRNA, and demographical & clinical (diagnosis age, ER status, PR status) data (72 features); (F) integration of copy number burden (CNB), tumor mutation burden (TMB), demographical & clinical (diagnosis age, ER status, PR status) data (5 features); (G) integration of mRNA, miRNA, CNB, TMB, and demographical & clinical (diagnosis age, ER status, PR status) data (74 features).


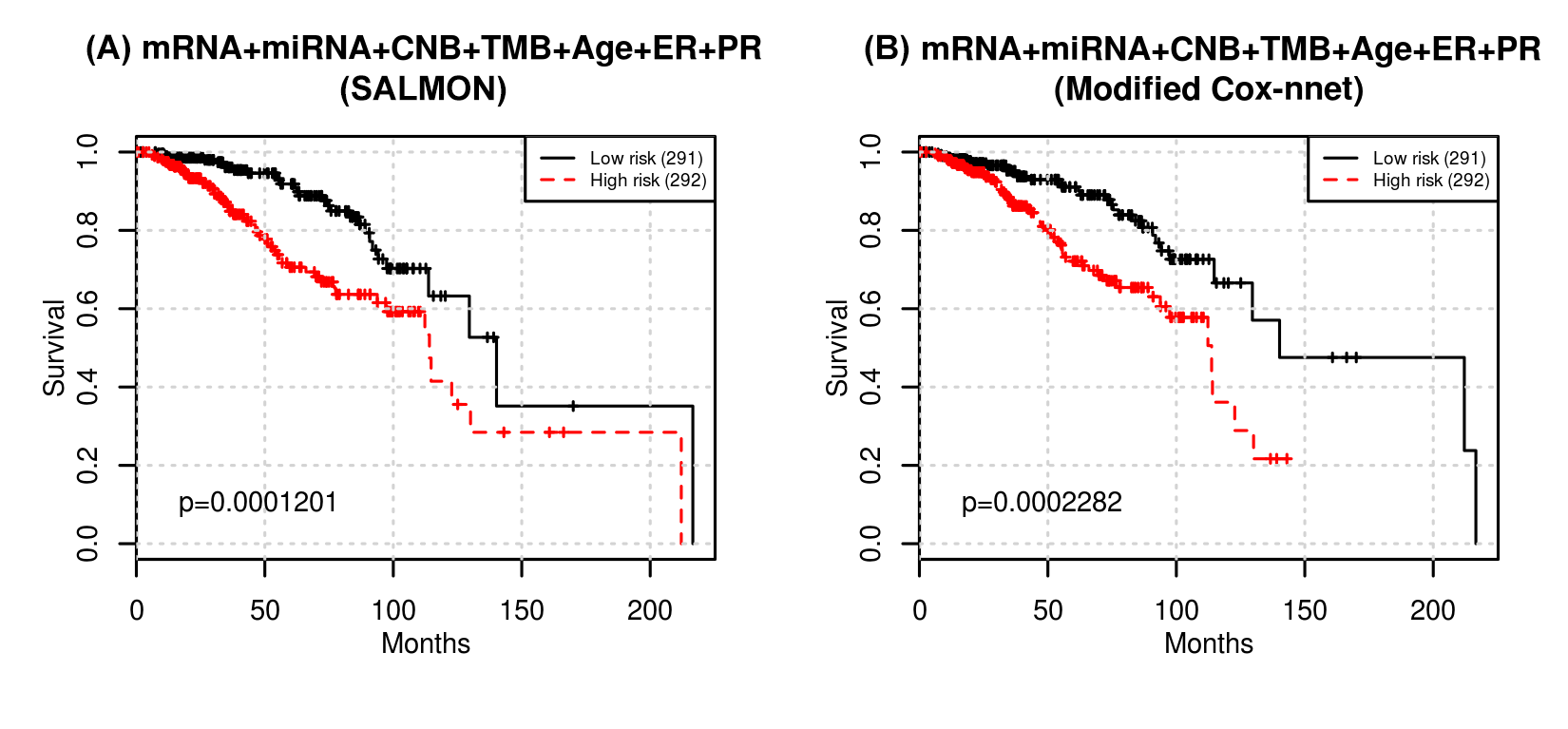


Figure S2. Performances comparison in terms of the p-value of the log-rank test between SALMON (A) and modified Cox-nnet (B) (Ching et al., 2018) with all omics data as inputs.


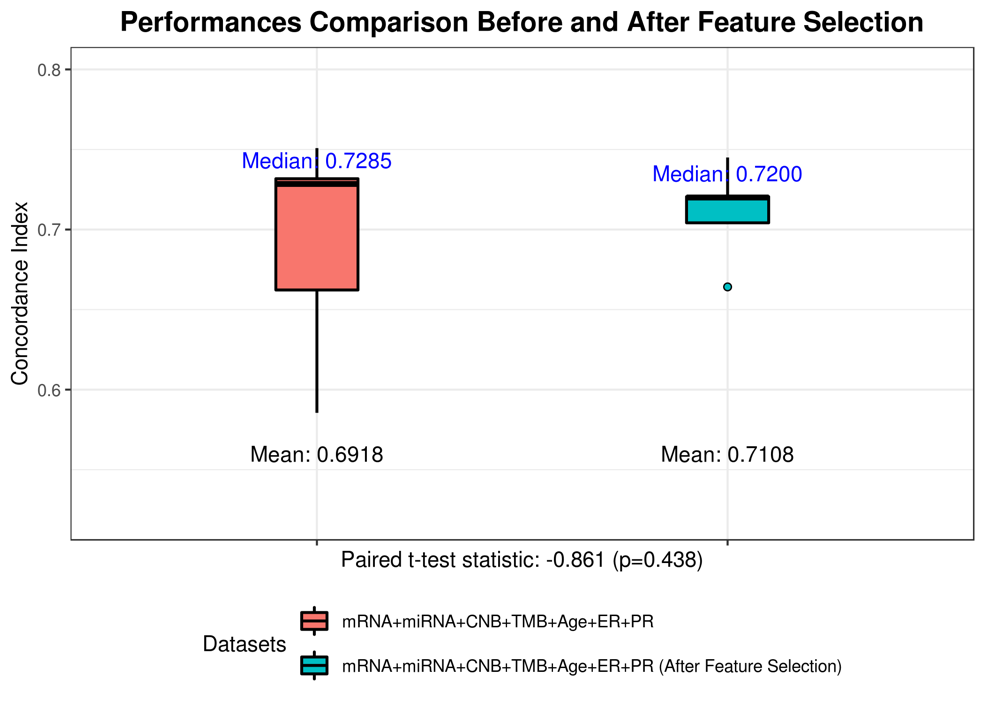


Figure S3. SALMON’s performance comparison of using all 74 multi-omics features and using selected 33 features (which their medians result in decrements to the concordance index in Figure 3). Selected 33 features are with ID 72, 74, 13, 47, 5, 36, 51, 19, 33, 29, 53, 20, 58, 66, 15, 16, 34, 70, 31, 42, 60, 11, 18, 71, 2, 10, 43, 44, 9, 32, 56, 62, 68 in Figure 3, where 24 of them are from mRNA co-expression modules, 5 of them are from miRNA co-expression modules, other 4 features are copy number burden (CNB), tumor mutation burden (TMB), diagnosis age, and progesterone receptors (PR) status. Concordance index before feature selection: median = 0.7285, mean = 0.6918; after feature selection: median = 0.7200, mean = 0.7108. Paired t-test statistics = -0.861 (p-value = 0.438).


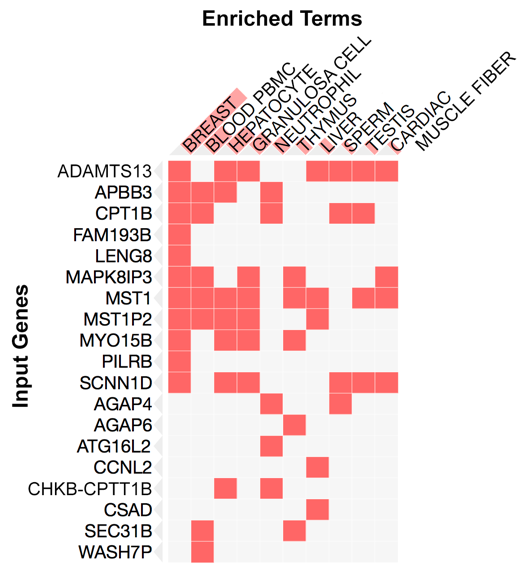


Figure S4. Enriched ARCHS4 Tissues terms with mRNA co-expression modules 13. nearly one third of genes (11 out of 36) in this module are associated with breast cancer bulk tissue (p-value=1.867E-03). Results generated from Enrichr (Kuleshov et al., 2016).


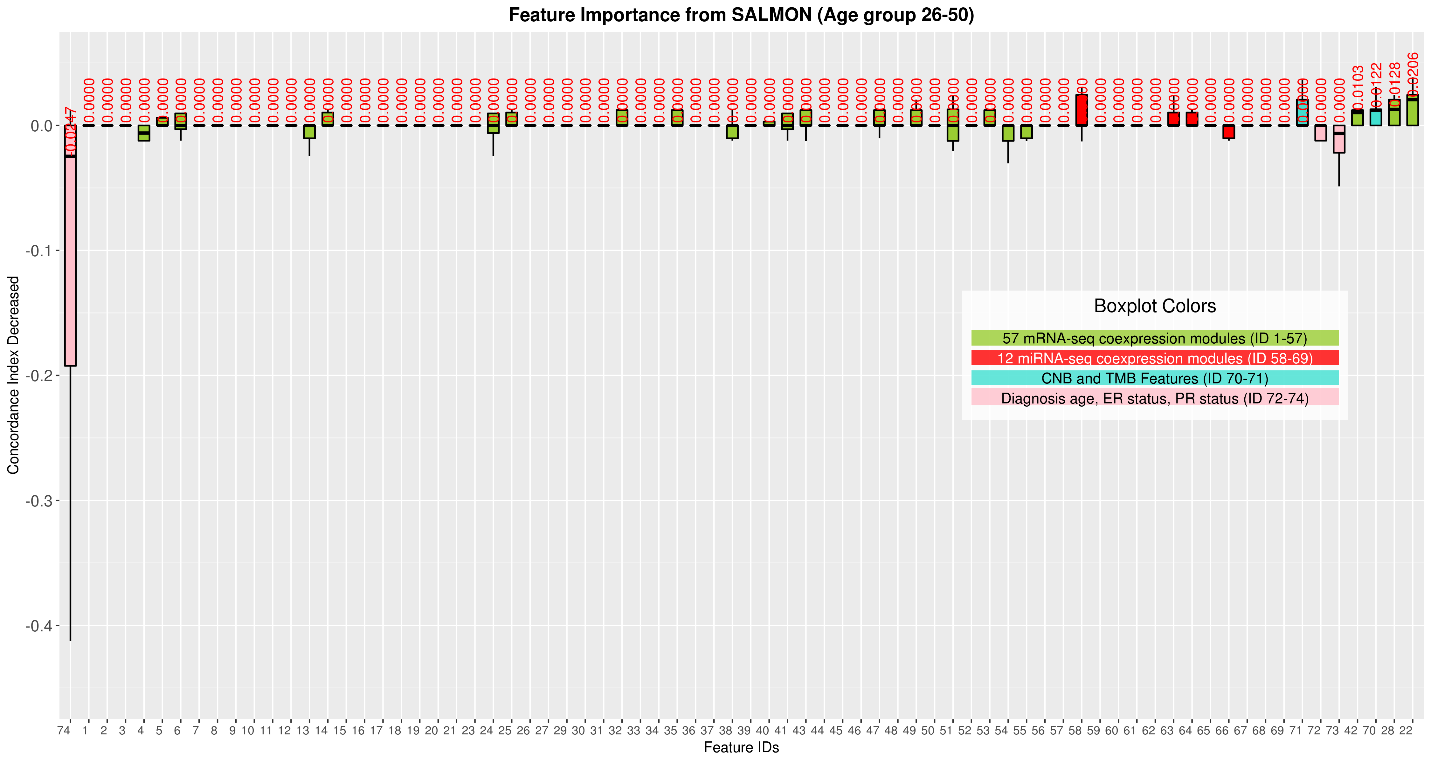


Figure S5. Feature importance with the diagnosis age in range 26-50, evaluated by the decrease of concordance index, sorted based on median values. Boxplots in Green: 57 mRNA co-expression module features (ID from 1 to 57); boxplots in red: 12 miRNA co-expression module features (ID from 58 to 69); boxplots in turquoise: copy number burden (CNB) and tumor mutation burden (TMB) features (ID from 70 to 71); boxplots in pink: demographical and clinical features (ID from 72 to 74).


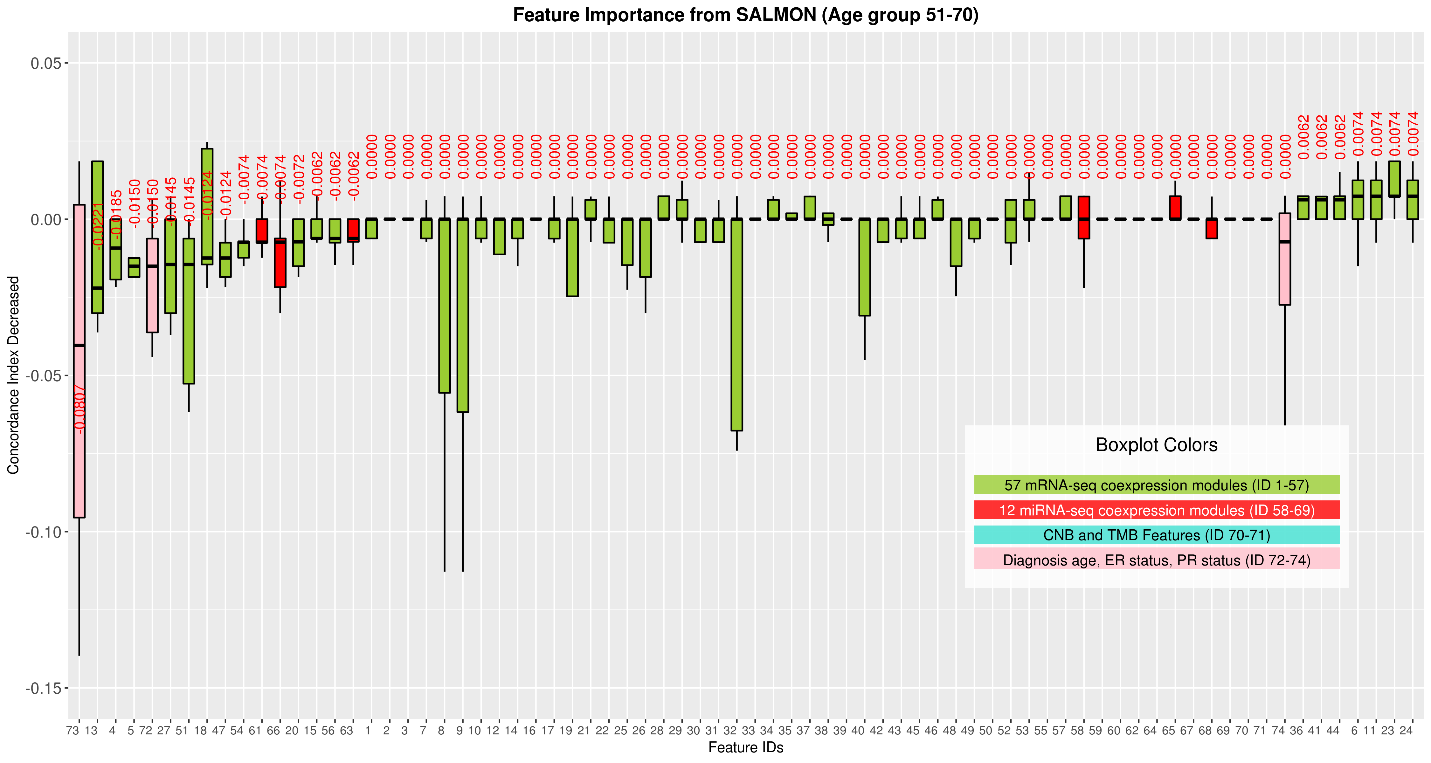


Figure S6. Feature importance with the diagnosis age in range 51-70, evaluated by the decrease of concordance index, sorted based on median values. Boxplots in Green: 57 mRNA co-expression module features (ID from 1 to 57); boxplots in red: 12 miRNA co-expression module features (ID from 58 to 69); boxplots in turquoise: copy number burden (CNB) and tumor mutation burden (TMB) features (ID from 70 to 71); boxplots in pink: demographical and clinical features (ID from 72 to 74).


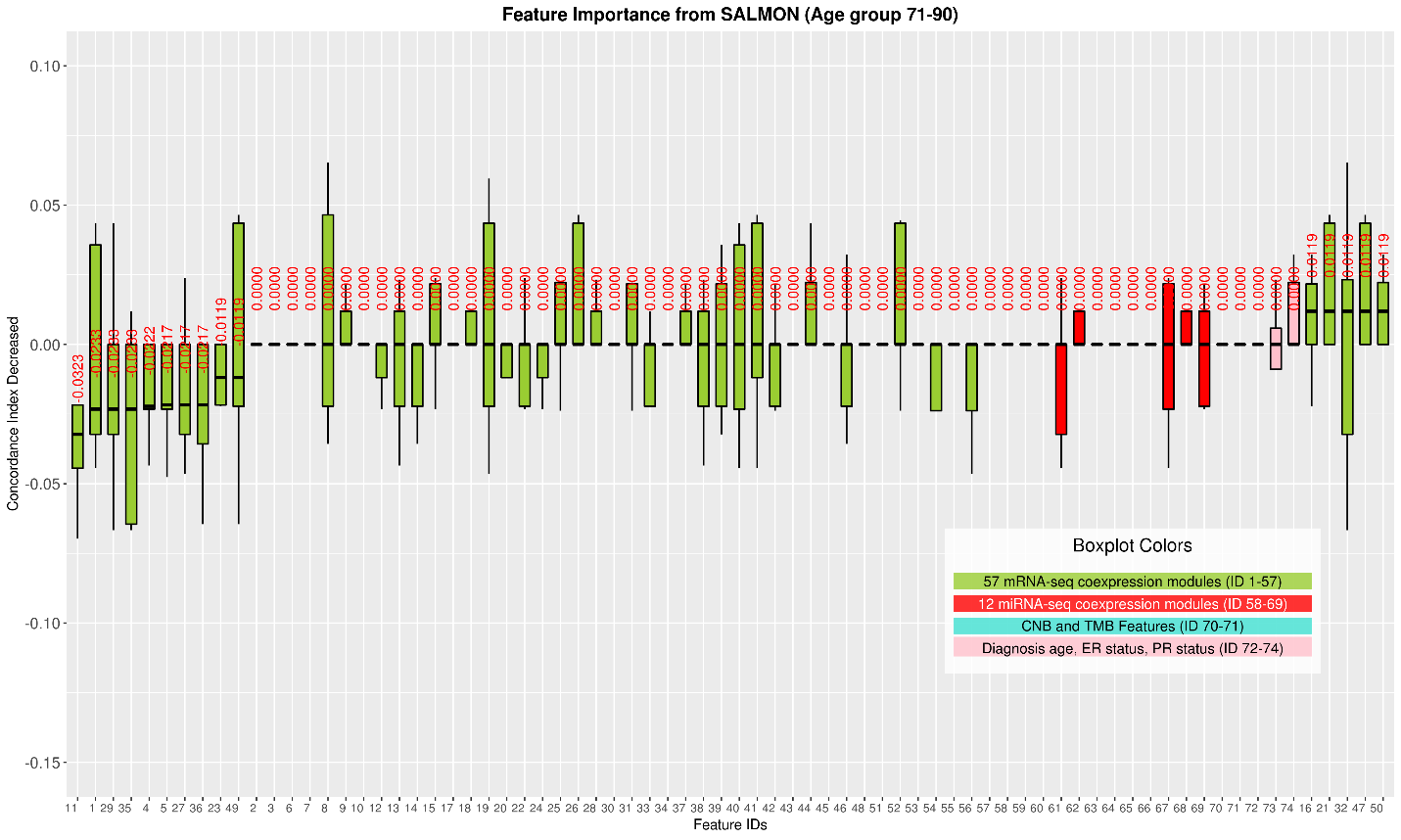


Figure S7. Feature importance with the diagnosis age in range 71-90, evaluated by the decrease of concordance index, sorted based on median values. Boxplots in Green: 57 mRNA co-expression module features (ID from 1 to 57); boxplots in red: 12 miRNA co-expression module features (ID from 58 to 69); boxplots in turquoise: copy number burden (CNB) and tumor mutation burden (TMB) features (ID from 70 to 71); boxplots in pink: demographical and clinical features (ID from 72 to 74).

**References**

Ching, T., Zhu, X., and Garmire, L.X. (2018). Cox-nnet: An artificial neural network method for prognosis prediction of high-throughput omics data. *Plos Computational Biology* 14(4). doi: 10.1371/journal.pcbi.1006076.

Kuleshov, M.V., Jones, M.R., Rouillard, A.D., Fernandez, N.F., Duan, Q., Wang, Z., et al. (2016). Enrichr: a comprehensive gene set enrichment analysis web server 2016 update. *Nucleic Acids Res* 44(W1)**,** W90-97. doi: 10.1093/nar/gkw377.
